# Influence of PHA Substrate Surface Characteristics on the Functional State of Endothelial Cells

**DOI:** 10.3390/jfb14020085

**Published:** 2023-02-02

**Authors:** Galina A. Ryltseva, Alexey E. Dudaev, Natalia G. Menzyanova, Tatiana G. Volova, Natalia A. Alexandrushkina, Anastasia Yu. Efimenko, Ekaterina I. Shishatskaya

**Affiliations:** 1 Department of Medical Biology, School of Fundamental Biology and Biotechnology, Siberian Federal University, 79 Svobodnyi Av., 660041 Krasnoyarsk, Russia; 2 Institute of Biophysics SB RAS, Federal Research Center “Krasnoyarsk Science Center SB RAS”, 50/50 Akademgorodok, 660036 Krasnoyarsk, Russia; 3 Basic Department of Biotechnology, School of Fundamental Biology and Biotechnology, Siberian Federal University, 79 Svobodnyi Av., 660041 Krasnoyarsk, Russia; 4 Institute for Regenerative Medicine, Medical Research and Education Center, M.V. Lomonosov Moscow State University, 119192 Moscow, Russia

**Keywords:** biodegradable polymers, polyhydroxyalkanoates (PHAs), copolymers, films, properties, cell cultures, mechanotransduction, surface coating, fibronectin

## Abstract

The needs of modern regenerative medicine for biodegradable polymers are wide and varied. Restoration of the viability of the vascular tree is one of the most important components of the preservation of the usefulness of organs and tissues. The creation of vascular implants compatible with blood is an important task of vascular bioengineering. The function of the endothelial layer of the vessel, being largely responsible for the development of thrombotic complications, is of great importance for hemocompatibility. The development of surfaces with specific characteristics of biomaterials that are used in vascular technologies is one of the solutions for their correct endothelialization. Linear polyhydroxyalkanoates (PHAs) are biodegradable structural polymeric materials suitable for obtaining various types of implants and tissue engineering, having a wide range of structural and physicomechanical properties. The use of PHA of various monomeric compositions in endothelial cultivation makes it possible to evaluate the influence of material properties, especially surface characteristics, on the functional state of cells. It has been established that PHA samples with the inclusion of 3-hydroxyhexanoate have optimal characteristics for the formation of a human umbilical vein endothelial cell, HUVEC, monolayer in terms of cell morphology as well as the levels of expression of vinculin and VE-cadherin. The obtained results provide a rationale for the use of PHA copolymers as materials for direct contact with the endothelium in vascular implants.

## 1. Introduction

The lack of a perfectly hemocompatible synthetic material is still one of the major problems in the field of biomedical engineering [1]. In reconstructive vascular technologies, this lack of currently available materials prevents successful clinical applications of vascular implants such as small-diameter blood vessel prostheses, heart valves, and stents. The development of polymeric materials with improved compatibility with blood is an urgent task in modern research [2]. An important aspect of the hemocompatibility of vascular implants is their correct endothelialization. Dysfunction of the endothelial layer, as an internal component of the vascular wall, formed in contact with the biomaterial and actively in contact with blood, largely determines the development of thrombotic complications accompanying cardiovascular implantation [3]. An interesting strategy for improving the compatibility of blood with implant materials is the development of surfaces that promote the formation of healthy endothelium, thereby reducing the risk of thrombosis [4].

Experimental and clinical studies indicate the successful application of an approach that includes an analysis of the features of mechanochemical signaling and a directed modification of the topography of biomaterials used in reconstructive tissue engineering [5].

Engineering of biologically active surfaces in vascular reconstructions is associated with the study and creation of micro/nanostructured surface reliefs that mimic the characteristics of the extracellular matrix and realize their activity in relation to cellular elements through mechanochemical signaling systems [6,7,8,9,10,11,12,13,14,15,16,17,18,19,20]. Morphological and dimensional characteristics of relief elements, as well as the degree of their ordering, can selectively influence the processes of differentiation, proliferation, and apoptosis [21,22,23,24,25,26,27,28,29,30].

It is known that the consistency of the endothelial layer on the surface of a biomaterial in vitro depends on the morphological features of the surface relief and the physical and mechanical characteristics of the material [31,32,33,34,35,36]. Substrate topography has been demonstrated to be a key regulator of many endothelial cell functions, including adhesion, morphology, migration, proliferation, and gene expression in vitro [37,38,39,40,41,42].

The flexibility and elasticity of the material are important factors for cell growth. The search for new materials with certain topographic features is an urgent task in biomedical engineering.

Polyhydroxyalkanoates (PHAs) are bacterial polyesters that are biodegradable, bioinert, and hemocompatible and are being actively studied in the field of tissue engineering [43,44]. It can be argued that this class of biological polyesters, being about fifty years younger in biomedicine than materials based on polylactide, is their worthy rival, having several serious advantages, including the nature of the biodegradation process (PHAs degrade in vivo with the formation of intermediates found in the human body [45,46]). The uniqueness of this class of materials is that it is possible to obtain copolymers of various compositions with different physical and mechanical properties. It is known that the inclusion of such monomers as 4-hydroxybutyrate (4HB), 3-hydroxyvalerate (3HV), and 3-hydroxyhexanoate (3HHx) affects the physical and mechanical properties [47,48,49]. Features of the microstructure and surface relief of PHA samples of different monomeric composition depend on the inclusion of these monomers, which affect the biological properties [50,51]. A thermoplastic polyester, poly-3-hydroxybutyrate (P3HB), has almost the same properties as polypropylene. Pure P3HB is considered too brittle for many applications, but its copolymers with monomers such as 4HB, 3HV, and 3HHx have superior mechanical properties compared to pure P3HB homopolymers. These copolymers are also being actively studied in the field of tissue reconstruction [52,53,54].

The native extracellular matrix (ECM) is a complex system consisting of many macromolecules with different biochemical and physical properties that play a key role in cell behavior [55]. Adsorption of ECM biomolecules onto the surface of a material is a promising approach to recreate the cellular microenvironment. In vascular implants, this treatment promotes proper endothelialization of the implants, thereby reducing their thrombogenicity. Extracellular matrix proteins, including fibronectin, have been shown to improve the adhesion and retention of cells to materials. These proteins provide the best support structure for many cell types, and fibronectin is a suitable support structure for endothelial cells [56].

The aim of this research is to study the features of endothelialization of the surface of PHA films of various compositions with different topographic features in vitro using human umbilical vein endothelial cells (HUVEC). The study of the surface reliefs of PHA polymers of various compositions, which is for the evaluation of endothelialization processes, will be carried out for the first time. In our research, we studied polymer films of four different compositions with various structural characteristics. Our hypothesis is that the composition of the polymer and the surface structure of PHA films, in combination with the use of fibronectin (Fn) as an ECM component, can affect the parameters of adhesion and proliferation of endothelial cells. The development of surfaces based on biodegradable polymer materials that are bioactive with respect to the endothelium can potentially contribute to the development of new strategies in the bioengineering of vascular implants.

## 2. Materials and Methods

### 2.1. Preparation of PHA Film Substrates

The films were obtained from samples of polyhydroxyalkanoates (PHA) of various chemical compositions, which were synthesized in the culture of the *Cupriavidus necator* B106-46 strain at the Laboratory of Biotechnology of New Biomaterials of the Siberian Federal University (Russia) [57]. It is poly-3-hydroxybutyrate (P3HB) homopolymer and three types of copolymers: poly-3-hydroxybutyrate-co-3-hydroxyvalerate (P3HB3HV), with 3HB:3HV monomer content as 85.0:15.0 mol%; poly-3-hydroxybutyrate-co-4-hydroxybutyrate (P3HB4HB), with 3HB:4HB as 64.5:35.5 mol%; and poly-3-hydroxybutyrate-co-3-hydroxyhexanoate (P3HB3HHx), with 3HB:3HHx as 62.0:38.0 mol%.

Polymer recovery from cell biomass was performed in two stages. First, lipids and fatty acids were removed using ethanol, and then the polymer was extracted with dichloromethane. The dichloromethane extracts were pooled and evaporated twice using an R/210V rotary evaporator (Büchi AG, Flavil, Switzerland). Then, the polymer was precipitated with hexane, 1:2. Polymer content in the residual mass was determined using a 7890A gas chromatograph equipped with a 5975C chromatograph-mass spectrometer (Agilent Technologies, Santa Clara, CA, USA). The polymer was redissolved in chloroform several times and precipitated using isopropanol or hexane to purify it. The resulting polymer was dried at 40 °C.

The purity and composition of the polymer were determined by chromatography of methyl esters of fatty acids after methanolysis of polymer samples using a 7890A gas chromatograph equipped with a 5975C chromatograph-mass spectrometer (Agilent Technologies, Santa Clara, CA, USA).

The chemical composition of PHA (the set and ratio of monomers in copolymers) was determined by chromatography of methyl esters of fatty acids after methanolysis of cell biomass using a 7890A chromatograph-mass spectrometer (Agilent Technologies, Santa Clara, CA, USA) equipped with a 5975C mass detector. 

The molecular weight and molecular weight distribution of PHA were determined using gel permeation chromatography with a refractometric detector on a DB-35MS column (Agilent Technologies 1260 Infinity, Waldbronn, Germany). Weight average molecular weight (Mw), number average molecular weight (Mn), and polydispersity (Ð) were measured using polystyrene standards (PS) (Agilent Technologies, Santa Clara, CA, USA). X-ray experiments were performed to determine the crystallinity of PHA specimens employing a D8 ADVANCE diffractometer (Bruker, AXS, Karlsruhe, Germany) equipped with a VANTEC fast linear detector. The degree of crystallinity (Cx) was calculated as a ratio of the total area of crystalline peaks to the total area of the radiogram (the crystalline + amorphous components).

The PHA films were produced using the solution-cast method. PHA samples of different chemical compositions were dissolved in choroform (3% solution) to obtain a homogeneous solution using magnetic stirring with heating to 35 °C. Afterwards, the solution was poured onto defatted glass, followed by drying for 48 h in a vacuum desiccator (Labconco, Kansas City, MO, USA) until the solvent was completely exhausted. The films were kept for at least 3 days in a fume hood, after which, using a template, discs were cut to the required size. The thickness of the films was measured with a LEGIONER EDM-25-0.001 electronic digital micrometer (Legioner, Shanghai, China). 

For sterilization, PHA samples were incubated three times with ethanol for 10 min, washed with sterile distilled water, irradiated with UV radiation for 60 min, and incubated in a culture medium for rehydration. 

### 2.2. Analysis of Surface

The surface relief of the films was studied using scanning electron microscopy TM4000 (Hitachi, Minato-ku, Tokyo, Japan). The samples were sputter coated with platinum (at 25 mA for 60 s) using the Leica EM ACE200 (Leica, Vienna, Austria) to obtain a good-quality image. The ImageJ image processing program was used to analyze SEM images of films of various chemical compositions, considering the total number of pores and their sizes.

The surface roughness of the films was determined using atomic force microscopy (AFM) DPN 5000 in semicontact mode (NanoInk, Skokie, IL, USA.). The AFM data were processed, and a statistical analysis of the images was performed using the free and open-source software Gwyddion (2.51) for image analysis and processing.

The energy properties of the surfaces were studied on a DSA-25E drop shape analyzer (Krüss, Hamburg, Germany) using DSA-4 software. To calculate the free surface energy and its dispersion and polar components (mN/m), the method of Owens, Wendt, Rabel, and Kaelble was used [58,59,60].

### 2.3. Endothelial Cell Culture and Immunocytochemistry

Human umbilical vein endothelium (HUVEC) were obtained from the biobank of the Institute for Regenerative Medicine of M.V. Lomonosov Moscow State University (Russia); collection ID: MSU_HUVEC. The cells were cultured under standard conditions in EGM-2 (Lonza, Basel, Switzerland) with the addition of an antibiotic-antimycotic at 37 °C in a 5% CO_2_ incubator. Cells were passaged at 70% confluency. For the assay, cells were used between passages 3 and 5.

All PHAs films have been coated by a selected concentration of fibronectin (Fn) solution with a 60 min incubation process to select the optimal concentration (experimental variant). In various experiments, original (uncoated) samples of polymer films from certain types of PHA or of tissue culture polystyrene (TCPS) coated with 1% gelatin (Sigma, Pompano Beach, FL, USA.) or fibronectin (IMTEK, Moscow, Russia) were used as controls. 

The sterile samples of films were placed into 96-well culture plates (Corning Costar, New York, NY, USA.). HUVEC were seeded at 5 × 10^3^ cells/cm^2^. The biocompatibility of the investigated materials has been assessed according to the mortality and survival of cells. Briefly, after 24 h of incubation, a solution consisting of Hoechst 33342 (Thermo Fisher Scientific, Waltham, MA, USA.) and an ethidium homodimer (Sigma-Aldrich, St. Louis, MO, USA) were added directly to the wells for the visualization of living and dead cells and were incubated for 5 min (Hoechst 33342 marked the nuclei of all cells; ethidium homodimer—dead cells only). Imaging was performed by the Cytation 5 multimode plate reader (BioTek, Winooski, VT, USA) with appropriate Gen5 software (BioTek, Winooski, VT, USA). The system performed panoramic imaging with fluorescence signals from cells (18 fields of view for each well with their subsequent stitching). The number of living cells is expressed as the difference between the number of cells labeled with Hoechst 33342 and ethidium homodimer.

To assess morphology, HUVEC were fixed with glutaraldehyde after 24 h of incubation, then counterstained with 1% OsO_4_ solution for an hour, and then the samples were dehydrated using alcohols. Before microscopy, the samples were sputter-coated with platinum. Samples were analyzed using scanning electron microscopy TM4000 (Hitachi, Minato-ku, Tokyo, Japan).

To assess the morphology of HUVEC, cells were selected after 24 h of incubation and fixed with glutaraldehyde, then counterstained with 1% OsO4 solution for an hour, and then the samples were dehydrated with alcohols. The samples were sputtered with platinum and analyzed using a scanning electron microscope TM4000 (Hitachi, Minato-ku, Tokyo, Japan).

VE-cadherin was used to characterize intercellular contacts and the mechanosensitive vinculin protein, which is a part of the multimolecular focal adhesion complex and forms a bidirectional link between the actin cytoskeleton and cell-matrix adhesive contacts, were visualized in nascent adherens junctions. Briefly, cells were washed with phosphate buffered saline (PBS) to remove protein residues, fixed with 4% paraformaldehyde at room temperature, and washed with PBS. After that, the cells were permeabilized with 0.2% Triton-X in PBS for 10 min, washed with PBS, and non-specific binding sites were blocked with a 1% bovine serum albumin (BSA) solution containing 10% of normal goat serum in PBS. The cells were then incubated with solutions of monoclonal antibodies to vinculin and VE-cadherin (Abcam, Cambridge, UK) in a 1% BSA solution in PBS for 16 hours at 4 °C. The cells were then washed with PBS, and the nuclei were labeled with DAPI (Invitrogen, Carlsbad, CA, USA). Fluorescence was read using a Leica DMI8 (Leica, Vienna, Austria) inverted microscope with appropriate LAS X software.

All post-acquisition image processing was conducted with ImageJ.

### 2.4. Statistics

The statistical analysis of the results was performed by conventional methods, using the standard software package of Microsoft Excel. Each experiment was performed in triplicate. Standard deviations were found. The statistical significance of the results was determined using Student’s *t*-test (significance level: *p* ≤ 0.05).

## 3. Results and Discussion

The physicochemical characteristics of PHA samples that determine the main properties of the material are given in Table 1. The maximum degree of crystallinity for the P3HB homopolymer is 75%. The inclusion of 3HV, 3HHx, and 4HB monomers into the polymer chain leads to a decrease in the degree of crystallinity of the copolymers. The predominance of the amorphous phase over crystalline is more pronounced in P3HB4HB (22%). The average molecular weight of the polymers also varied depending on the monomeric composition, being the maximum for P3HB (920 kDa) and the minimum for P3HB3HHx (486 kDa).

The analysis of the surface morphology of PHA films revealed differences in the relief profile and porosity. These parameters affect cell adhesion. It is known that smoother surfaces have less adhesiveness than materials with a rough surface [61,62].

**Figure 1:**
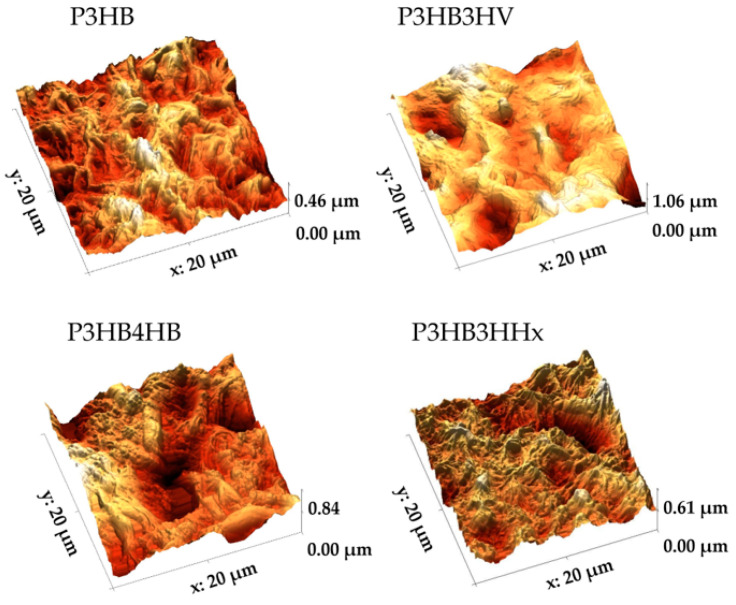
AFM images of PHA polymer films of various chemical compositions.

Using atomic force microscopy, the samples were found to have differences in the parameters of nano-roughness, which determine protein adhesion, cell growth, and attachment. The dependence of the surface relief on the composition of polymers was determined (Table 2, Figure 1). The maximum values of the arithmetic mean roughness (Sa) and root mean square roughness (Sq) of the surface have the sample P3HB4HB—290.31 and 370.60 nm, respectively. The P3HB film had the lowest values of these parameters, while the analogous values for the P3HB3HHx and P3HB4HV samples were higher by 40–70 nm. The values of the parameter expressing the surface roughness according to the selected five maximum heights and pit depths (Sz) differed between P(3HB) and its copolymer with 4HB by 1.8 times—1255.8 and 2321.2 nm, respectively. The high value in the case of P3HB4HB is due to the presence of deep pores in the sample. The Sz values for P3HB3HV and P3HB4HB were close and approximately 350 nm higher than the minimum value [50].

**Figure 2:**
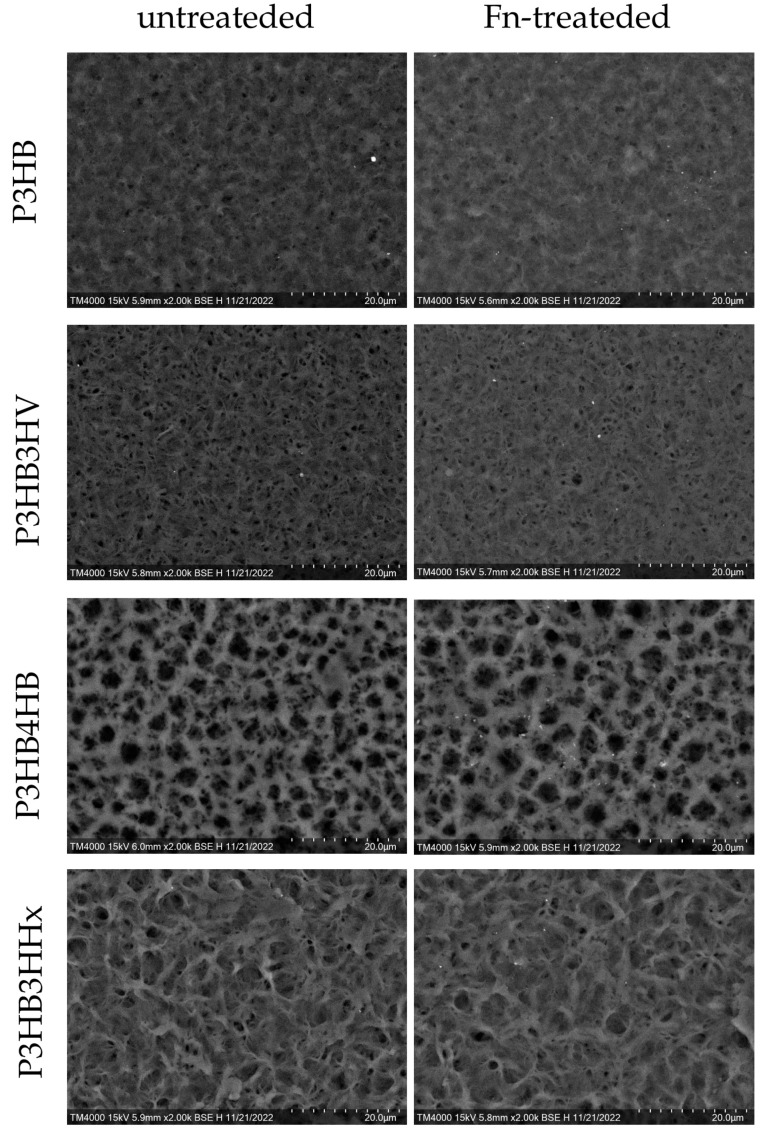
SEM images of PHA films of various chemical compositions before and after fibronectin coating.

The films were similar in thickness (60.3 ± 5.8 µm) but considerably different in morphology. The ImageJ image processing program was used to analyze SEM images of the films with different chemical compositions, taking into account the total number of pores and their sizes (Table 3, Figure 2). AFM data on roughness correlate with the number of pores on the surface of polymer films. The P3HB film had a smoother surface compared to the copolymer samples, with a small number of small pores and an average pore size of 0.052 ± 0.032 µm. The pore diameter of the P3HB3HV copolymer sample was close (0.056 ± 0.029 µm), but their total area was higher (8.686%). The P3HB3HHx sample had a folded structure with numerous small pores with an average diameter of 0.081 ± 0.07, and their area was the lowest (1.248%). The highest porosity is characteristic of the P3HB4HB film; their diameter (0.272 ± 0.255 µm) and total area (17.470%) were an order of magnitude greater than those of other films.

It was found that the area of pores on the surface of films obtained from copolymer samples decreased after application and adsorption of fibronectin by almost 1.5 times, and somewhat more significantly (2 times) for the homopolymer sample. It is important to note that the ratios of the average pore size and area between samples of all compositions did not change after treatment with Fn, which argues in favor of the assumption of a uniform distribution of Fn on all samples. As in the works of Xiang-HuaQu [63], in our case, the formation of an Fn layer on the surface of the samples contributed to the smoothing of the surface relief.

It is known that the hydrophilicity of materials is a key factor influencing the interaction between cells and the matrix. The hydrophobicity of the surface promotes the adhesion of proteins, which contribute to the active cell-substrate interactions [64,65]. If the surface is too hydrophobic, ECM proteins, including Fn, are adsorbed in a denatured state, and their geometry becomes unsuitable for cell binding [66]. In our study (Table 4), Fn adsorption increased the wettability of all PHA samples. A slight decrease in the contact angle of approximately 6° was noted, except for P3HB4HB. The homopolymer had a maximum water contact angle of 90.7° after treatment with Fn, remaining the most hydrophobic of all PHAs studied. P3HB3HV, P3HB4HB, and P3HB3HHx had similar values: 78.9 ± 4.21, 77 ± 5.38, and 78.5 ± 4.71, respectively. The experimental data are confirmed by the studies of other authors. Ya-WuWang [67] investigated the effect of P3HB and P3HB3HHx hydrophilicity on the growth of the L299 cell line. Better adhesion was reported on lipase-treated PHA surfaces with a contact angle of about 60–70°, compared to a surface treated with a combination of lipase and hyaluronic acid with a contact angle of about 50–60°. Bumgardner et al. [68] studied the surface coated with chitosan (contact angle 76.4°) and the titanium surface (contact angle 32.2°). It was found that the attachment of cells to the first surface was better than that of pure titanium. 

An increase in surface energy, including its polar component, of 1–2 mN/m was also observed, which promotes the adhesion of ECM proteins, thereby improving cell attachment. In general, surface energy is a fundamental material property that can influence protein adsorption. Low surface energy and hydrophobicity generally correlate with increased protein adsorption, enhanced conformational changes in adsorbed proteins, and irreversible protein adsorption. Thus, the effect of surface energy on cellular response observed in the studies [64] was probably an indirect effect, where surface energy dictated protein adsorption and conformation, which subsequently determined a specific cellular response. Scott B. Kennedy et al. confirmed that surface energy can affect cell adhesion and proliferation through Fn, which is the dominant factor determining the response of cells to materials. A study by B. Majhy et al. [69] showed that average surface energies lead to efficient cell adhesion, growth, and proliferation. An increase in surface energy leads to the formation of stronger cell-ligand bonds, which leads to increased growth and proliferation. When the critical surface energy is exceeded (more than 70 mN m^−1^), cell growth and proliferation slow down.

It should be noted that endothelial cells are very demanding on the structure and quality of the scaffold surface; they attach poorly and practically do not proliferate on many known materials, including cultural plastic [70]. Therefore, for the successful cultivation of endotheliocytes, it is very important to provide conditions for their attachment to the surface of the scaffold and optimal subsequent proliferation. Various materials are used as coatings to improve the adhesive properties of the surfaces of cell carriers, including gelatin, collagen, polysaccharides, and fibronectin [15,16,19]. Experience has shown positive results from the use of gelatin coatings for the routine use of this protein for culturing various cells, including endothelial cells [71,72]. An increase in the adhesion properties of surfaces is also provided by the application of hydrophilic coatings or treatment with chemical reagents.

It should be noted that the studied polyhydroxyalkanoates, despite their high biocompatibility and prospects for various biomedical applications, including cell technologies, are characterized by high hydrophobicity; they do not hydrolyse or swell in liquids. Thus, the contact angles of 3-hydroxybutyric acid homopolymers are up to 90 °C and higher; for copolymer films, these values are slightly lower (Table 4). Therefore, in order to prepare polymer film scaffolds from PHA, it is recommended to soak them for 2–3 h in a sterile saline solution before seeding the cells [73].

Increasing the wettability of PHA product surfaces is also possible using physical methods, for example, hydrogen peroxide plasma treatment [74], argon or ammonia plasma [75], as well as laser irradiation [76]. However, the use of very labor-intensive physical methods requires special equipment and proven processing modes.

Therefore, to improve the adhesive properties of the surface of PHA films, the effectiveness of the use of a fibronectin coating was studied in the present work. In this case, it was necessary to find out how the adhesion of endothelial cells is affected by different concentrations of Fn covering film matrices from PHA of different chemical compositions.

**Figure 3:**
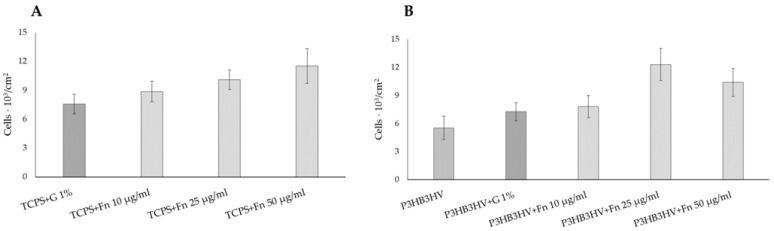
The amount of live adherent HUVEC cells on the surface of the sample: (**A**)—HUVEC on TCPS+Fn; and (**B**)—HUVEC on films P3HB3HV+Fn.

At the first stage, the effect of fibronectin coating on the adhesion and development of HUVEC cells was studied when they were grown on TCPS with Fn coating (Figure 3A). The TCPS coated with 1% gelatin was used as a control since this is used in the routine cultivation of HUVEC [77]. Since the purpose of this experiment was to test the effects of Fn coating and not to grow a significant number of endothelial cells, the duration of observation was 24 hours. Similar durations of HUVEC culturing were described by other authors. Thus, when studying the development of endothelial cells on porous polyethylene glycol membranes that differ in microstructure, the duration of the experiments was also 24 hours [78,79]. A comparative study of the adherence of endothelial cells to TCPS for 24 hours, coated with gelatin and fibronectin (Figure 3A), showed a statistically significant difference in the case of using fibronectin at a concentration of 25 and 50 µg/mL. The number of live HUVEC cultured on TCPS+Fn increased commensurately with protein concentrations. The data indicate the expedient further use of the Fn coating of PHA films.

A similar positive effect of the Fn coating was recorded when fibronectin was deposited on the PHA films. The P3HB3HV sample was used to test the coating of PHA films with different concentrations of fibronectin during the 24 h of cultivation of HUVEC using Fn solutions at concentrations of 10, 25, and 50 μg/mL. On the example of a sample obtained from the P3HB3HV copolymer (Figure 3B), the effect of the Fn concentration on the number of living HUVEC cells in a 24 h culture was studied relative to the initial uncoated film from this copolymer (control). A sample of the P3HB3HV copolymer coated with a 1% gelatin solution was used as the second control in this experiment. The lowest cell count was on the original, uncoated film of this PHA copolymer. The effect on the number of HUVEC cells of gelatin coating and Fn at a concentration of 10 mg/mL on the number of living cells was comparable. Increasing the concentration of fibronectin to 25 and 50 mg/mL significantly increased the number of living endothelial cells, with the best result obtained using an Fn concentration of 25 mg/L. Therefore, in the following experiments, a coating of a 25 mg/mL fibronectin solution was used.

**Figure 4:**
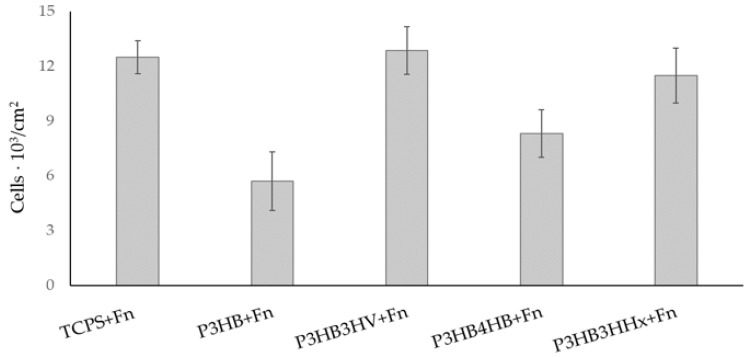
The amount of adherent HUVEC cells on the surface of the samples of PHA films coated with fibronectin at a concentration of 25 mg/mL.

Figure 4 illustrates the results of the influence of the PHA+Fn films and the number of living endothelial cells during their 24 h cultivation on films of various compositions coated with Fn. 

Experimental samples with Fn at a concentration of 25 µg/mL significantly increased cell proliferation on all PHA films. The smallest number of living cells was noted in the case of the P3HB sample; in the case of P3HB4HB, there were slightly more cells. The amount of HUVEC in the case of P3HB3HV and P3HB3HHx increased by almost 2 times and was comparable with the TCPS-Fn results. In general, PHAs of various compositions modulate cellular behavior. The highest number of living cells was on the films of the P3HB3HV copolymer (12.8 ± 1.3 10^3^/cm^2^), and this is comparable with the result obtained with TCPS+Fn as a control. In general, all Fn-coated PHA copolymer films are suitable for culturing endothelial cells.

The results obtained are consistent with the few available publications on the effect of cell matrices from PHA for culturing endothelial cells. The use of various variants of functionalization of the surface of Fn materials is also noted in the literature. Xiang-HuaKu et al. [63] obtained P3HB3HHx films that were coated with a 10 mg/mL Fn solution, which increased the number of adherent cells by 1.5 times compared to uncoated PHA material. These data are consistent with the work of Pompe T. et al. [80] on the modification of P3HB and P3HB4HB films with an aqueous solution of NaOH, NH_3_-plasma, or H_2_O-plasma and Fn. The strength of Fn interaction with the PHA surface significantly decreased after treatment with alkali; however, the adsorbed protein at a concentration of 50 µg/mL on untreated PHA films significantly improved attachment to the surface of HUVEC cells. Xiang-Hua Qu et al. [81] noted that the metabolic activity of HUVEC is higher on P3HB3HHx compared to the P3HB homopolymer and its copolymer with 3HV. Samples from P3HB3HHx also had the lowest hemolytic activity compared to other samples; for example, hemolysis rates were 3 times higher for the P3HB homopolymer, and almost 2 times higher for the copolymer with 3HV.

Thus, the surface morphology of PHA polymer films, which is characterized by the size and number of pores on the surface as well as roughness, correlates with the indicators of viability and adhesion of endothelial cells. Thus, a homopolymer with an almost smooth surface and a small relative pore area, as well as its copolymer with 4HB, which has the largest number of wide pores, have lower adhesiveness, which is confirmed by the number of living cells on these films. The surface structure of the P3HB3HV and P3HB3HHx samples, which have pores of 47 ± 3 and 236 ± 20 nm in size with a pore area of 5.9 and 12.4%, respectively, facilitates the adhesion of HUVEC cells and corresponds to the data in the control (TCPS-Fn). Gaborski et al. [78,79] demonstrated that the pores on the membrane can disrupt the interaction of the cell with the substrate, which is consistent with our results. D. Narayan and S. S. Venkatraman [61] studied the effect of pore size on PLLA and PLGA endothelialization. Small pores in the range of 5–20 µm instead of pores of 20–90 µm have been shown to be more preferable for culturing HUVEC. Sang Jin Lee et al. [82] noted that cells adhered well and distributed well on polycarbonate (PC) membranes with micropore diameters of 0.2–1.0 µm. Such cells had a spindle cell morphology. However, most cells with larger micropore sizes (3.0–8.0 µm in diameter) showed a more spherical cell shape with fewer filopodia and lamellipodia, indicating a decrease in the adhesiveness of the material. Zahra Allahyari et al. [62] confirmed in their review that small pores at the nanoscale promote the strongest cell-substrate interactions, while the largest pores at the micron scale prevent these interactions. It is also known that smooth surfaces have weaker adhesion than the surface of a material with moderate porosity. The work of Ahmad Fikri bin Anwar Fadzil et al. [83] showed that it is more problematic for cells to adhere to a smooth surface because filopodia do not move away from the cells.

**Figure 5:**
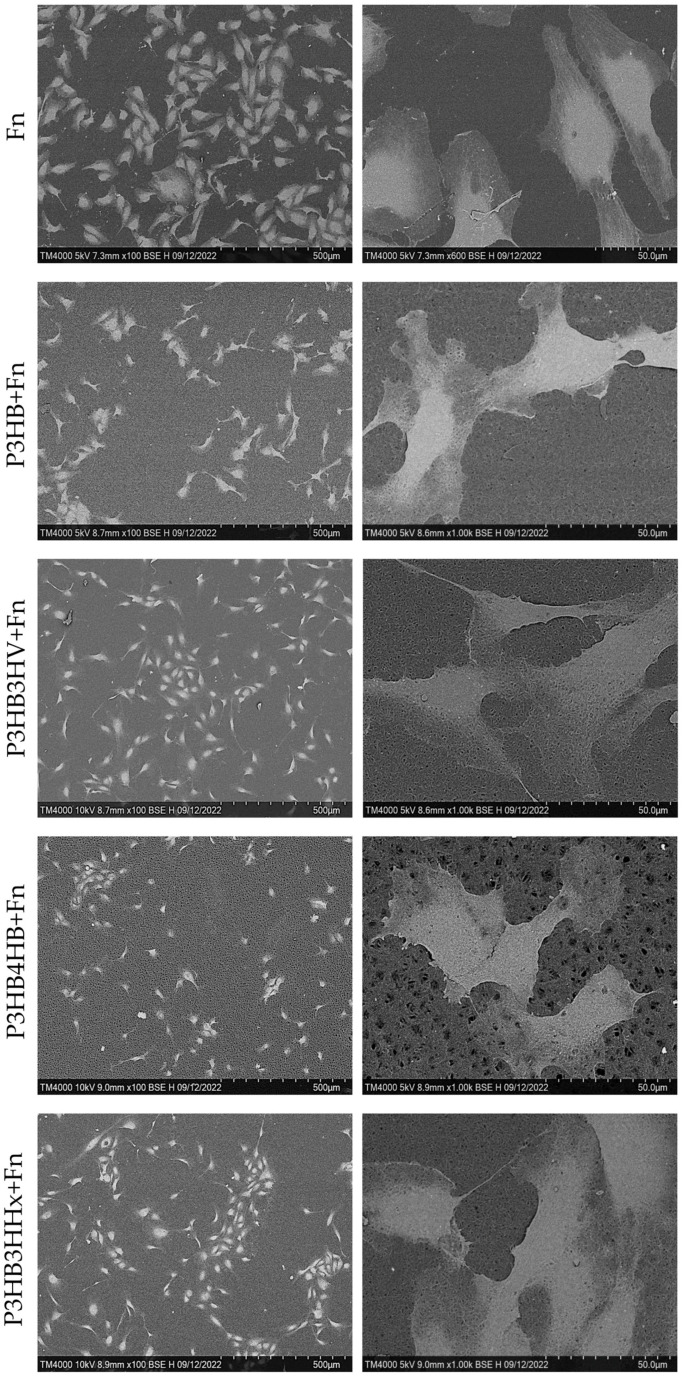
SEM-Image of adherent HUVEC cells on the surface of the samples of PHA-films.

Further, using scanning electron microscopy followed by image analysis using ImageJ software, it was found that the cells on the studied samples are smaller with a larger length-to-width ratio compared to TCPS-FN (Figure 5). It should be noted that the cells on the homopolymer and on its copolymer with 4HB are less flattened, which is consistent with the results of the cytotoxicity and adhesion assessments. Obviously, this is due to the smaller number of cell adhesion points on the surface of the material—both the smoothest of all homopolymers and the most porous P3HB4HB. Cells that formed a monolayer had a cobblestone shape typical for endothelial cells, while single cells were more elongated and had a spindle morphology, indicating their mobility. At a small distance between single cells, contact of the lamellipodia is observed, which is the initial stage of the formation of adherens junctions (AJ).

**Figure 6:**
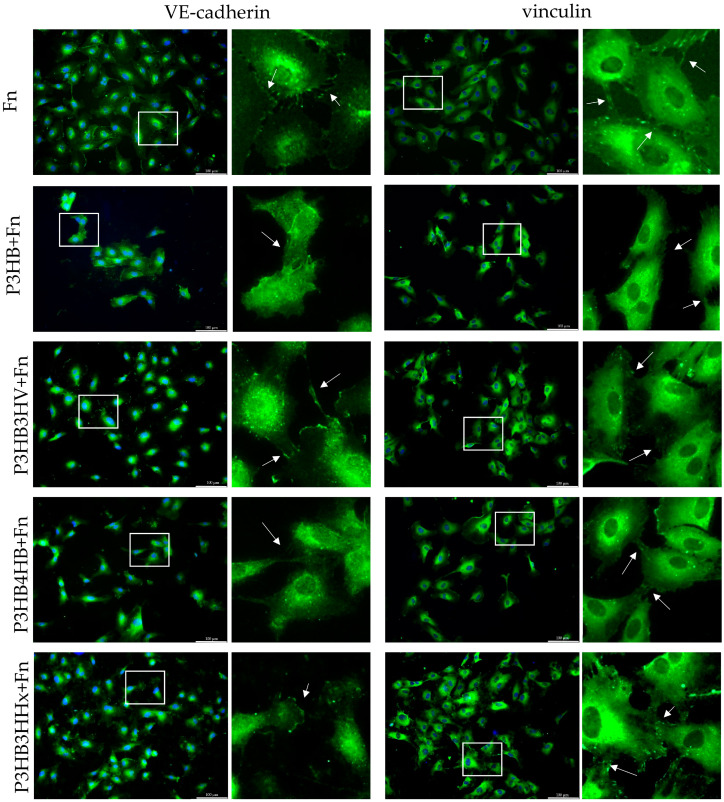
Distribution of VE-cad and vinculin in adherent HUVEC cells on the surface of PHA film samples. Marker 100 µm. The general plan is on the left; an enlarged image of a highlighted white rectangle is on the right.

Julien Brevier et al. [84] confirmed that physical contact between adjacent cell lamellipodia promotes the recruitment of AJ proteins such as VE-cadherin (VE-cad). Indeed, a similar situation was observed during immunohistochemical staining with anti-VE-cad in our study. In the case of P3HB3HV, P3HB3HHx, and control samples, redistribution of VE-cad into the contact zones along the cell periphery was noted (Figure 6). On the contrary, intracellular localization of VE-cad was observed on the homopolymer sample, which indicates a low migration of this protein into adhesive contacts. Aggregation of VE-cad in adjacent lamellipodia of neighboring cells was also practically absent in the case of P3HB4HB, which indicates a less active interaction between cells during cultivation on samples of this composition [85,86].

In the present study, the participation of this protein in maintaining the endothelial barrier during changes dictated by the cytoskeleton in mechanochemical interactions with polymer samples with different surface profiles is considered [87,88,89]. Data from Stephan Huveneers [90] show that there is a vinculin-dependent mechanosensory interaction between cadherins that mediates adequate remodeling of endothelial junctions to promote, for example, transendothelial migration of leukocytes to the site of injury or infection to reduce the inflammatory response. Thus, when studying intercellular contacts, it can be noted that the maximum number of vinculin binding sites is observed on the P3HB3HHx sample at the points of contact between the lamellipodia of neighboring cells. Additionally, the minimum number of binding sites for this protein is observed in cells on a homopolymer sample, on which vinculin was distributed throughout the cytoplasm without active binding sites (Figure 6).

Thus, PHA films obtained from polymers of various monomer compositions differed significantly in physical properties that affect the adhesion and distribution of cells: the ratio of crystalline and amorphous regions (degree of crystallinity), surface roughness, hydrophobic–hydrophilic balance, the number, and the size of pores on the surface of the films. The present study demonstrated that none of the types of PHA films had a negative effect on human umbilical vein endothelial cells. Surface functionalization with fibronectin significantly improved the biocompatibility of the material with respect to HUVEC. The results of this study show that P3HB3HV and P3HB3HHx films are the most favorable for HUVEC cultivation. Such films have average values of hydrophobicity, roughness, and crystallinity in relation to all the studied samples, which makes these polymers promising for creating vascular implants.

## 4. Conclusions

The results obtained in this study show that films of biodegradable polyhydroxyalkanoates of various monomer compositions with adsorbed fibronectin are biocompatible with respect to human umbilical vein endothelial cells. It has been shown that treatment of PHA films with Fn at a concentration of 25 μg/mL makes the surface smoother, reducing the number and area of pores. This treatment also slightly increases the hydrophilicity and surface energy of the surface, which improves adhesion and cell growth. It can be concluded that HUVEC adhere and proliferate on all PHA film samples. It has been shown that the surface structure has an effect on cell adhesion and proliferation. On the smoother surface of P3HB and on the surface of P3HB4HB, which has large, deep pores, a slightly smaller number of attached cells was noted. A greater amount of HUVEC with active distribution of VE-cad and vinculin in the contact zones of lamellipodia was noted on samples P3HB3HHx and P3HB3HV, which indicates the consistency of intercellular and adhesive contacts of the monolayer formed on the surfaces of PHA samples. The fact that various types of cells react differently to surface topography is of significant importance in the research of the development of implants, since it is expected that a functionally consistent endothelial monolayer formed on the surface of PHA materials will have a significant effect on the hemocompatibility of the device and thereby reduce the risks of thrombotic complications during the implantation of these devices into the bloodstream.

## Figures and Tables

**Table 1:** The characteristics of PHA samples.

Samples	Average Molecular Weight (Mw) kDa	Polydispersity (Ð)	Degree of Crystallinity (Cx) %
P3HB	920	2.5	78
P3HB3HV	690	2.8	65
P3HB4HB	660	3.6	22
P3HB3HHx	486	3.7	52

**Table 2:** The roughness of PHA samples.

Samples	Root Mean Square Roughness (Sq), nm	Arithmetic Mean Surface Roughness (Sa), nm	Peak-to-Valley Height (Sz), nm
P3HB	180, 26	142, 83	1255, 77
P3HB3HV	254, 24	206, 92	1594, 61
P3HB4HB	370, 60	290, 31	2321, 24
P3HB3HHx	222, 69	172, 62	1677, 55

**Table 3:** Characterization of pores on the surface of PHA samples.

Parameters	P3HB	P3HB3HV	P3HB4HB	P3HB3HHx
	Untreated			
area, %	1.519	8.686	17.470	1.248
average size, µm	0.052 ± 0.032	0.056 ± 0.029	0.272 ± 0.255	0.081 ± 0.070
	Fn-treated			
area, %	0.785	5.920	12.364	0.756
average size, µm	0.035 ± 0.010	0.047 ± 0.028	0.236 ± 0.200	0.064 ± 0.047

**Table 4:** The wettability of PHA samples.

Parameters	P3HB	P3HB3HV	P3HB4HB	P3HB3HHx
	Untreated			
Water contact angle (°)	94.5 ± 3.7	85.3 ± 1.7	72.2 ± 7.2	83.2 ± 3.2
Surface energy (mN/m)	35.6 ± 0.3	38.6 ± 0.5	46.2 ± 1.2	36.7 ± 0.5
Dispersion component (mN/m)	34.7 ± 0.2	35.7 ± 0.4	39.2 ± 0.7	32.5 ± 0.4
Polar component (mN/m)	0.9 ± 0.1	2.9 ± 0.1	7.0 ± 0.5	4.3 ± 0.2
	Fn-treated			
Water contact angle (°)	90.7 ± 2.8	78.9 ± 4.2	77.0 ± 5.4	78.5 ± 4.7
Surface energy (mN/m)	36.5 ± 0.6	39.6 ± 0.7	43.7 ± 1.6	38.8 ± 0.6
Dispersion component (mN/m)	34.9 ± 0.5	34.2 ± 0.5	38.4 ± 1.2	32.8 ± 0.3
Polar component (mN/m)	1.6 ± 0.1	5.5 ± 0.2	5.2 ± 0.4	6.0 ± 0.3

## Data Availability

Not applicable.
